# Child Rights during the COVID-19 Pandemic: Learning from Child Health-and-Rights Professionals across the World

**DOI:** 10.3390/children10101670

**Published:** 2023-10-09

**Authors:** Eva Jörgensen, Laura Wood, Margaret A. Lynch, Nicholas Spencer, Geir Gunnlaugsson

**Affiliations:** 1 Faculty of Sociology, Anthropology and Folkloristics, School of Social Sciences, University of Iceland, Sæmundargata 2-6, 102 Reykjavík, Iceland; geirgunnlaugsson@hi.is; 2 Department of Sociology, Lancaster University, Bailrigg, Lancaster LA1 4YU, UK; laura.wood@lancaster.ac.uk; 3 Department of Paediatrics, King’s College, Strand, London WC2R 2LS, UK; malynchuk@gmail.com; 4 Division of Mental Health and Wellbeing, Warwick Medical School, University of Warwick, Coventry CV4 9JD, UK; nick.spencer@warwick.ac.uk

**Keywords:** qualitative research, COVID-19, child health professionals, convention on the rights of the child, social sciences, child health, child advocacy, policymaking

## Abstract

The COVID-19 pandemic underscores the importance of a child rights-based approach to policymaking and crisis management. Anchored in the United Nations Convention on the Rights of the Child, the 3P framework—provision, protection, and participation—forms the foundation for health professionals advocating for children’s rights. Expanding it with two additional domains—preparation and power—into a 5P framework has the potential to enhance child rights-based policies in times of crisis and future pandemics. The study aimed to (1) gather perspectives from child health-and-rights specialists on how children’s rights were highlighted during the early phase of the pandemic in their respective settings; and (2) evaluate the usefulness of the 5P framework in assessing children’s visibility and rights. A qualitative survey was distributed among child health-and-rights professionals; a total of 68 responses were analysed in Atlas.ti 9 from a multi-disciplinary group of policymakers and front-line professionals in eight world regions. As framed by the 5Ps, children’s rights were generally not safeguarded in the initial pandemic response and negatively impacted children’s health and wellbeing. Further, children lacked meaningful opportunities to raise their concerns to policymakers. The 5P framework holds the potential to shape an ethical child rights-based decision-making framework for future crises, both nationally and globally.

## 1. Introduction

With the adoption of the United Nations Convention on the Rights of the Child (UNCRC) [1], nation-states’ moral obligations towards children, as fostered in the 1924 Geneva Declaration and the earlier Rights of the Child in 1959, became legally obligatory for authorities. The new convention provided governments with guidelines on how to consider children as human rights holders within areas of law and policymaking [2] and as agents with a voice in matters about their welfare [3]. The shift towards a human rights lens saw children as subjects instead of objects who do not need to be *given* rights; instead, they *have* them [4]. Consequently, the UNCRC became the first human rights treaty to combine civil, political, economic, social, and cultural rights without creating a subdivision or hierarchical structure, viewing all areas as interrelated and equally important [4].

There is a lack of clarity regarding the underlying values that inform decision-making in child health and social policy, for example, how the social status of children is perceived within society [5], how to make health policy child-focused [6] and garner children’s voices [7], or address their specific needs due to conditions such as disability [8]. According to Li et al. [9], children are among eleven vulnerable social groups significantly impacted by government interventions worldwide. As a result, the COVID-19 pandemic has raised questions about the status of children as right-bearing citizens, as stated in the UNCRC. The pandemic’s onset resulted in a swift transformation in the social and political environment concerning children’s rights, most notably regarding school closures [10]. Ying Tan et al.’s [11] review highlights children’s institutional and cognitive or communicative vulnerability as they could not respond to government decisions regarding school closures, which would often be enforced without much notice. Furthermore, official information channels often failed to reach them as the information was not sensitive to their abilities to understand. Additionally, Lygnegård, Thell, and Sarkadi [12] call attention to the importance of information dissemination tailored to children’s and adolescents’ levels of comprehension. Their study underscores the importance of considering them as active participants and decision-makers in matters vital to them.

Farmer [13] has shown the importance of equity measures in global health policy and epidemiological settings and argued that individual health conditions are entangled within a wider, multifaceted web of power structures. Singer’s biocultural theory of syndemics posits that epidemics are synergistically related [14], where, as a result of social forces, pre-existing illnesses are exacerbated by a new condition, as exemplified by the HIV epidemic. Similarly, COVID-19 has been declared not only a pandemic but also a syndemic [15]. During the pandemic, children with pre-existing conditions, such as asthma, diabetes, or malnourishment, experienced exacerbated health outcomes due to additional stressors on the healthcare system [16]. For instance, their access to healthcare was reduced as children’s facilities were taken over to accommodate the overflow of adult patients, leading to delayed interventions or preventative treatment. This exemplifies how children’s pre-existing conditions interlocked with the pandemic to become a syndemic, causing further marginalisation and health inequalities. The COVID-19 pandemic has, therefore, accentuated the importance of equitable measures for children.

Professionals and researchers concerned with children’s right to health and wellbeing have been pivotal in providing insights into areas of concern within government administration, healthcare, and social care [17,18,19,20], regarding equitable measures for children and their participation [21], access to healthcare [22], and the increasing digital divide [23,24,25]. Additionally, advocates for children’s rights play a crucial role in representing children’s voices at higher levels of governance [26]. In that capacity, they consider the UNCRC a tool to advance children’s rights within governmental and administrative practices, assuming the responsibility of safeguarding children’s rights [27].

Members of the International Society for Social Pediatrics and Child Health (ISSOP) and the International Network for Research on Inequalities in Child Health (INRICH) have a longstanding interest in working towards the betterment of children’s rights worldwide [28]. To document how children’s rights were affected by COVID-19, a qualitative survey was conducted with the aim to gather perspectives from child health-and-rights specialists across continents on how children’s rights had been highlighted during the early phase of the pandemic in their respective settings.

### The UNCRC and Its Domains

The UNCRC views children as rights-holding citizens and seeks to address and safeguard their fundamental rights, irrespective of geographical location or socioeconomic background, and promote their inclusion in government policy. To uphold the treaty’s comprehensive and holistic perspective and enhance its clarity, Hammarberg [29] suggested a three-part grouping of the articles into the so-called “three P’s”: provision, protection, and participation. Lansdown [30] later expanded the 3Ps to represent the importance of participation in child rights. The 3P framework is a conceptual paradigm encompassing the essential concepts outlined in the child rights articles. It delineates intentional and strategic components of research, practice, action, and advocacy for children and young people. Thus, adopting a child rights-based approach to policy based on the core principles, as Goldhagen et al. [26] suggest, can “address the inequities, laws, services, and budgetary allocations that advance or undermine the realisation of children’s rights”.

The evolving ideological and political context around children’s rights necessitates a re-evaluation of the concepts outlined in the UNCRC. Therefore, to better comprehend the multiple domains of the 3P framework and its potential as an evaluative instrument for children’s rights, it is imperative to examine the contexts in which these rights are genuinely implemented and distinguish them from instances when they are only tokenistic gestures. Such reassessment shifts the treaty’s role from merely an ideological instrument to a social and political significance framework, thereby enhancing its practical application [31].

Building on the original 3P framework, Moore, Melchior, and Davis [32] created the additional categories of *prevention* and *perception*, suggesting the “5P” framework to reflect better the nuanced realities of children and young people with disabilities. Cole-Albäck [33] later expanded the 3P framework with the domains of *promotion* and *power* to become an analytical tool regarding children’s rights, yet not calling it a 5P framework. Recently, Byrne and Lundy [34] suggested reconsidering the fundamental principles for an effective child rights-based approach in policymaking. Their framework offers a 6P structure: principles/provisions of the UNCRC; the process of children´s rights impact assessment; participation of children and young people; partnership to secure joined up working; public budgeting to ensure that the resources are in place for implementation; and publicity to make policies known to children and young people.

With the unfolding events of COVID-19 and the subsequent impacts on children worldwide, members of ISSOP and INRICH suggested the application of an expanded framework of 3Ps by adding two more domains to the arrangements of the UNCRC articles, namely *preparation* and *power* (Table 1). As such, the proposed 5P framework considers adequate *provision* for children’s health and developmental needs, *protection* from harm and exploitation, and valued *participation* in health, wellbeing, and life matters. The additional *preparation* suggests that state officials proactively plan for children’s health for the present and future generations. *Power* addresses the genuine inclusion of children’s voices and respect for their authority as agents who can illicit changes needed in matters about them.

Applying the proposed 5P framework (Table 1), the study’s primary aim was to gather perspectives from child health-and-rights specialists across continents on how children’s rights were highlighted during the early phase of the pandemic in their respective settings. A secondary aim was to evaluate the usefulness of the 5P framework in assessing children’s visibility and rights in this phase of the pandemic.

## 2. Materials and Methods

### 2.1. Study Design and Settings

**Figure 1:**
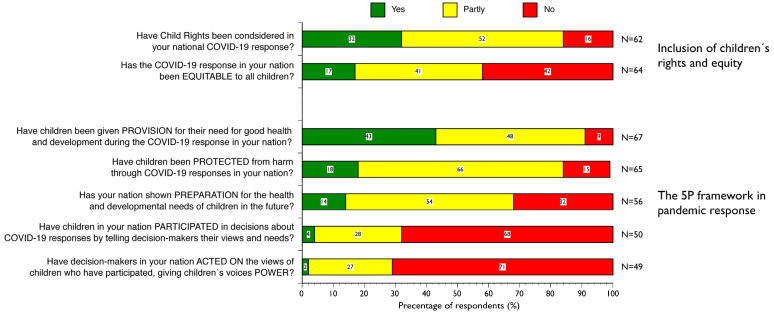
Response of the participants on children’s rights and equity during the COVID-19 pandemic and on the five main domains (5Ps) of the United Nations Convention on the Rights of the Child (UNCRC). N = number of valid responses, excluding missing values.

The collaborative Northern Europe regional ISSOP/INRICH research team constructed an online survey with a three-point scale for initial probing. The scale was Yes, Partly, and No, with an option of I do not know. This three-point scale was complemented with an open-ended option for further elaboration (Supplementary Materials File S1). The participants were invited to answer questions about children’s status in their government’s COVID-19 emergency policy, especially regarding the domains of the 5Ps, i.e., provision, protection, participation, preparation, and power (Figure 1; Supplementary Materials File S1). The online survey platform SurveyMonkey was active from February to October 2021.

### 2.2. Participants

Participants were members of ISSOP, INRICH, and Child Healthcare Information for All (CHIFA) and working as child health-and-rights advocates worldwide. Participants were identified with purposeful non-probability snowball sampling aiming for diversity rather than representativity [35]. The objective was to capture the insights and experiences of child health-and-rights professionals in diverse global locations. Members of ISSOP, INRICH, and CHIFA disseminated the qualitative survey through their networks, deliberately recruiting multi-disciplinary participants from different countries. In total, 72 professionals responded, residing in 32 countries in eight different world regions: sub-Saharan Africa (5), North America (2), South America (5), Middle East (2), Asia (5), Oceania (1), Australia (1), and Europe (11); the countries for two responses are missing. Four answers were removed due to incomplete survey responses, leaving a total of 68 responses analysed.

### 2.3. Analysis

The answers were coded in two phases using Atlas.ti. software (version 9.1.7). The first author (E.J.) performed the first level of open coding informed by grounded theory, which allowed the coding process to be data-driven and draw out the views and experiences of the participants. The relationship between the codes was then examined with axial coding. E. J. and the second author (L.W.) discussed and refined the codes via selective coding to identify central themes [36,37]. Based on the grounded theory coding, the second author (L.W.) performed the second coding stage and grouped the codes in line with the domains of the ‘5P’ framework, allowing the grounded theory approach to inform new fields and enabling the research team to draw insights from the entire survey. The coding process was iterative as it was discussed within the research team and refined by L.W. and E.J. until a consensus was reached.

### 2.4. Ethics

The University of Iceland Ethics Committee approved the study about the experiences of adolescents and health professionals of COVID-19 (No: SHV2020-024). The participants in the survey were adult child health-and-rights professionals who consented to participation by ticking a consent box before proceeding with the survey (Supplementary Materials). Participation was voluntary, with no personal identifiers.

To protect the anonymity of the participants, their geographical location is omitted in the results section. When relevant, the classification of countries as low-, middle-, or high-income is used as defined by the World Bank for 2021–2022 [38].

## 3. Results

The survey generated 68 responses for analysis from multi-disciplinary child health-and-rights professionals. All participants (*n* = 68) gave their responses to the three-point scale. Participants ranged from front-line professionals, including doctors, physicians, and paediatricians (65%), nurses (9%), psychologists (3%) and social workers (4%), to researchers (4%) and policymakers (6%), and other/unspecified (9%).

Two overarching themes address how participants viewed the rights and status of children and young people during the initial responses to the pandemic as it appeared worldwide. The initial theme considers their observations regarding the inclusion of children’s rights in their government’s emergency intervention and whether that response was equitable (Figure 1). The latter theme focuses on the 5Ps surrounding children’s rights, i.e., participation, protection, provision, preparation, and power, and how they were addressed during the initial months of the COVID-19 emergency response (Figure 1).

### 3.1. Inclusion of Children’s Rights and Equity

#### 3.1.1. Observation of Children’s Rights in Government Emergency Response

Just less than one-third of survey respondents reported that child rights had been considered in their government pandemic response (Figure 1). However, many thought children’s visibility could be drastically improved. One respondent from a high-income country described it as such:

[T]he rights of children are so weak and are subjugated to […] exercise […] the right and the protection of adults. Therefore, [the] […] global health crisis confirms the adult-centred development model and its priorities.

Across continents, participants reported that children’s rights placed low in the state’s emergency response, with all the focus on clinical-based interventions. Further, they highlighted a lack of knowledge and understanding about the importance of upholding children’s rights during critical times. The lack of awareness was manifested in assumptions such as children being at a lower clinical risk than adults exposed to COVID-19, which even turned into a certain ‘weaponisation’ of children in the media as carriers of the disease or as one participant from an upper-middle income country formulated it:

Children [were] invisible during the pandemic. We are now [looking into how] stereotypes around children [are created] from “nobodisation” […] to “weaponisation” [where] children are a threat. The rights of the child are not under debate. For the government, it is more important the right to health than other rights (i.e., education). This shows the lack of understanding of the rights perspective, considering that all rights are equally important, indivisible, and interdependent.

Many participants commented on the image of children as disease carriers. It had initiated fears in people, leading to ideas of children needing to be contained as disease spreaders, resulting in the reduced prioritisation of their rights to health, development, and wellbeing. Furthermore, while countries were prioritising the mitigation of the spread of the virus, other adult-centred arrangements were being prioritised, such as protecting the national economy.

Other aspects further contributed to the subjugation to the safeguarding of children’s rights, such as socioeconomic status and location, as a respondent from a lower-middle-income country described it:

Having schools closed for the whole pandemic to the majority of children has caused huge divides in access to online schooling. Many people experiencing poverty do not have access to the internet, so it has been [a] disaster for the children in the millions of lower-income households across the nation, both in a rural and urban context.

School closures prominently revealed the socioeconomic disparities among children. Not only was their right to education infringed but the welfare of those in lower-income households was notably compromised due to barriers in accessing digital education.

#### 3.1.2. Equity in Pandemic Responses

About two out of five of survey respondents felt their government’s response was inequitable (Figure 1). Still, for some, equity measures had to overcome barriers of pre-existing social situations, which was now exacerbated for families already living under vulnerable conditions. There were equitable aspects relating to the diversity of children’s needs, which had not been previously considered but had become more evident than before, for example, access to high-speed internet, school meals, and access to adults for protection. As all these elements are likely to meet and converge in the school system, inequities amongst children were prone to emerge via disruption to their daily school routine, requiring more reliance on online platforms for teaching. One respondent from a high-income country wrote:

During school closures, municipalities tried to provide food to those needing school meals but received little state support [so] charity activities and NGOs [non-governmental organisations] tried to fill the gap. Vaccination is officially free for everyone, but internet poverty makes it difficult to access both information and vaccine registration.

One respondent used the term ‘internet poverty’ in various answers to highlight the role of the internet in exacerbating pre-existing inequalities caused by educational disruption. Additionally, although access to digital solutions could be provided, not all children possessed the literacy required or had the assistance from adults to navigate the platforms needed for successful educational engagement.

### 3.2. The 5P Framework in Pandemic Response

#### 3.2.1. Participation of Children

Participants were asked about children’s participation level, and which measures the authorities had taken to ensure meaningful participation instead of tokenistic box-ticking. More than two-thirds of the survey respondents said children had not been part of the pandemic response decision-making process at all (Figure 1). A participant from an upper-middle-income country said:

Generally speaking, children were not part of the debate on their needs, priorities and desires. Few exceptions have to do with local efforts…that [got] consultation to get children’s voices.

Another participant from a low-income country described it as such:

For sociocultural and traditional reasons, children’s opinions are still not sufficiently taken into account in all domains, especially in COVID-19 contexts in which even adults are unaware, as we are in a society where the opinion of adults is more valued than the opinions of kids.

No respondent reported child rights-based approaches that welcomed and enabled participation of children and young people throughout the pandemic or participation that started early as the crisis emerged. Many respondents across various continents reported that there needed to be more government-led opportunities for children to participate meaningfully. Where this did occur, as noted in two high-income countries, such opportunities came relatively late after emergency measures were already in place. One noted that there were “several experiences where children and adolescents [were] asked about their opinions and suggestions by both public institutions (…) and private non-profit [organisations]. This has been increasingly done during the second wave”. The other participant said:

The impact on children was estimated to a limited degree regarding very few emergency measures. This was generally done after emergency measures had been developed and implemented…views of children should have been taken into account in their development…the Minister of Education…convened a meeting with children on emergency measures regarding limitations concerning schools and online teaching arrangements. This consultation came late and when emergency measures had been implemented. This consultation with children…was important…and should have been more extensive.

Furthermore, the participants reported that information on how to involve children as participants was ambiguous and vague, as stated by one of the participants:

Children[’s] participation is an area without progress. Some projects may exist from time to time, but nothing on a scale or at least recognised as essential. The issue is that there is no clear information on how to make it happen.

The quote echoes that most participants were unsure about children’s participation in their countries. Another participant in a high-income country reported that there were isolated examples of child participation in some settings; however, “…overall, children have not participated in decisions”. Instead, opportunities for children’s involvement came from NGOs or grassroot bodies providing children with local pandemic-related health education and social support. These initiatives also offered participation via surveys and hosting discussion platforms for children, allowing them to ask questions to policymakers.

Survey participants reported that later in the pandemic, there were examples of more significant efforts to communicate with children and young people about the situation. One participant from a high-income country described the lack of involvement of children and young people during the earliest stages of the pandemic. Still, as the pandemic continued, children’s participation grew:

During the third wave of the pandemic […], children have been more involved in creating information material appropriate for children. For example, there is a special kid-news program on national TV with children as presenters. Children have also been interviewed in the media and have been given the opportunity to direct questions to those in charge, e.g., on TV.

Therefore, as respondents claimed, children’s participation was generally not a part of the earliest stages of the pandemic. Still, as the pandemic evolved, respondents observed the importance of involving children in information dissemination or as stakeholders had grown more robust, if it did so at all. It depended on the country’s resources, such as the strength of the media and internet connectivity. In most cases, children’s opinions and wishes were carried out by adult advocates at the administrative level of governance.

#### 3.2.2. Protection of Children in a Pandemic Response

Just less than one out of five survey respondents reported that their government’s pandemic response had protected children (Figure 1). Many respondents observed that schemes with specific protective measures had been set in place by governments, such as food schemes, financial support for parents, and raising awareness towards the possibility of an increase in domestic violence. However, participants considered that the importance of other societal measures had suppressed children’s need for protection against domestic abuse. An example of this narrative is one from a high-income country that described how governments had put more emphasis on physical distancing despite reports about violence in children’s domestic and private spheres, the focus being on:

social (physical) distancing and increased hygiene measures [which are] designed to reduce viral spread and load. [However], there is an increase in reported child abuse cases, domestic violence, and cyberbullying. Children need better protection under the threat of increased domestic violence.

A participant from a lower-middle-income country also highlighted children’s need for further protection and the increase in domestic violence:

Some kids have been provided with good shelter at home. Some kids go without food because of reduced income… [We] need to protect children in the communities. There were increase [in] instances of child abuse… [We] need to work and empower parents and families…for them to continuously provide for the needs of kids…

The above responses echoed those of most participants, who described children facing barriers within the health and care system due to physical distancing and agreed that more actions were needed on a governmental and societal level to consider the secondary effects physical distancing had on children’s access to health and care. One participant from a high-income country claimed, “society has not done all it could to protect children”, referencing that a more holistic approach needs to be considered when children’s matters are at stake.

In cases where respondents mentioned socioeconomic measures, they highlighted cumulative disadvantages regarding children, families, and communities living under pre-pandemic poverty and economic stress that struggled to receive enhanced support. For children with learning difficulties, developmental conditions, and disabilities, especially those in special schools and with high support needs at home, reduction in access to professionals within and outside the school system, careers, and respite support was highly challenging. Minority communities, such as refugees, asylum seekers, and others with unstable immigration status, faced additional practical and language barriers plus complexities caused by stigma and fear of authorities. One high-income country participant mentioned that although information about the pandemic could be accessed in various languages, the information may “…nonetheless not reach some minority groups”, which meant additional barriers for children dependent on parental capacity to access and understand official information. A participant from the same country added that “migrant and refugee children and parents” might not seek information due to “fear of deportation”. One participant in a high-income country described that children in foster homes might not have “…been treated like other children” during the pandemic. The respondent did not elaborate further. However, descriptions and concerns about the increased risk of harm were prominent for most participants. Another report from a high-income country emphasised the need for improved information dissemination:

Some children have been kept at home from preschool due to frightened parents. There has been a lack of information and support for these families. There are signals that more children during the pandemic have been neglected or maltreated due to parents’ economic, mental, or abuse problems.

Where survey participants felt government support was lacking, some reported community-based or grassroots-based initiatives. It could be, as an example, within the context of a high-income country, a teacher carrying “packed lunch for kids who might need them” or an initiative shown by an NGO action that implemented mental health and psychosocial support to bridge the need where no child protection system exists. Welfare projects were generally reported to be scarce on the national level. One respondent from a lower-middle-income country wrote: “Every province is conducting programmes on COVID-19 psychological support. We [...] are implementing mental health and psychosocial support for 20,000 children and their families in [the region]”.

#### 3.2.3. Provision for Children’s Needs

Over two out of five survey respondents reported that governments had considered children’s needs in their pandemic responses (Figure 1). However, one respondent from a high-income country described there being “… a gap between a general provision in theory and practicable provision in the sense of delivery and reception”. The answer harmonises with the notion expressed by many respondents that although policymakers considered actions that aligned with children’s rights and needs, such intentions were not followed by action. The basic life needs of most children were met, such as shelter, nutrition, and sanitation, with many nations rapidly developing novel solutions such as cash transfers and food aid. However, many support systems, such as access to health facilities, often provided within the school grounds, were disrupted. For example, one participant from an upper-middle-income country wrote, “There [were] emergency programmes to provide food and cash transfers to the family”. A participant from another lower-middle-income country provided a more nuanced picture of the pandemic’s impact on children’s provisions, pointing out that while some children had good shelter at home, others went hungry. Their parents could not provide for their needs because of a lack of income when business was closed or open for a reduced number of hours. Further, some children lacked life skills, as described by a participant from a lower-income country “to navigate challenges in the communities over the COVID-19 period,” with many girls becoming pregnant. Nations with pre-existing, better-functioning health and health insurance systems demonstrated flexibility and the capacity to move to virtual consultations and other formats of health care. One respondent from a high-income country wrote:

The health insurance system is good [...]. The national budget officially covers 80% of the medical cost. And most local governments subsidise the remaining 20% cost [...]. During the pandemic, many parents refused to visit a paediatric clinic. So, the government flexibly agreed to cover medical care costs using telephone [short message services] and another remote system as public medical insurance.

A similar narrative was evident in an answer from another respondent, who even noted the benefits of the online health provision shift for children with complex needs that service providers had established during the pandemic. A participant from a high-income country suggested online consultations had become “rather welcome and may help develop different kinds of services”. As these families and their children often need frequent hospital visits, providers could continue to develop such digital solutions. Respondents from nations with pre-existing challenges to health and wellbeing provision for children and those experiencing catastrophic collisions of the pandemic with conflict, economic crisis, and electrical supply failure reported being extremely limited in their ability to respond to the evolving emergency. One participant from a lower-middle-income country said, as an example:

The pandemic disrupted all levels. This affected the care children receive in general, including vaccinations…The pandemic coincided with a terrible economic crisis, which made food and basic necessities harder to obtain. This also resulted in partial to complete loss of electric power, which made staying at home and studying “online” more difficult.

Other respondents commented on how parental fear created a barrier to children’s access to services despite being functional and available, such as attending clinical appointments; therefore, routine aspects of child health were negatively affected. A participant from an upper-middle-income country describes “immunisation” as having “fallen behind” with “parents reluctant to attend clinics for fear of infection”, leaving health and social sectors to catch up on the accumulated caseload.

#### 3.2.4. Preparation for the Future Needs of Children

Over a tenth of the survey respondents reported future preparation for children in their country (Figure 1). There needed to be more clarity regarding what would constitute preparation. However, in many instances, this was mentioned as mental health intervention or prevention; one participant from a high-income country observed that “some…funds [have been] dedicated for mental health projects”, although it remained unclear whether this was due to more influx or relocation of funds that already been allocated. Others described a visible impact on children and young people’s mental health; however, “…no preparation for future resources” was being made. Another respondent from a high-income country, on the other hand, had a more positive description of their government’s preparation plan:

[T]here are a number of initiatives which have been taken: a specific section in the national resilience plan services and programs for support to families—an initiative to strengthen social services in underserved areas—an initiative to strengthen the curricula of child professionals (health education, social …).

Several participants reported that national leadership had announced increased health budgets from now on and long-term public health action plans. Some did mention that previous staggered reduction of standard class sizes in schools may cause further problems in the future and additional burdens on resources as latent secondary impacts of the pandemic may arise. Some participants raised concerns that schools needed to be engaged in future preparedness plans for possible future pandemics. However, on other levels, governmental health budgets had already been increased on local and national levels, and many predicted the normalisation of online medical care and educational services to accommodate changes in need and the ability to help.

#### 3.2.5. Children’s Power

Only one survey respondent, from a high-income country, reported children having had the power to influence their nation’s pandemic response (Figure 1). Here, power signifies the authority of voice with indications of action informed by children in pandemic responses. Participants gave examples of the children’s ombudsmen and ministries that collected and mediated children’s opinions to the broader public. Such initiatives were mainly absent, as one participant from another high-income country stated: “There is no system for it”.

Generally, participants reported that their governments had no pre-existing structures to facilitate children’s participation in decisions, which would subsequently impact them. They characterised this by an associated lack of regard and dismissal of the value of children’s voices in navigating a national and global crisis. Respondents recorded that local organisations such as community groups, schools, and sports clubs provided meaningful opportunities for children to influence change. However, participants also described a discrepancy between theory and practice. Where children’s ministers, their ombudsmen, and advocates of a similar position were present, responses reflected limited efficacy in promoting children’s rights, voice, and power. One respondent from a high-income country wrote: “The national ombudsman for children and adolescents has taken the initiative to voice children’s views. There are several local experiences where this has also occurred. However, little real action has followed.”

The narrative of children’s voices not being included and acted on directly in practice was evident. Growing pressure from parents and civil society organisations to involve children in several nations led to limited action. In other cases, local professionals, including social paediatricians, heard children’s voices and then championed the children’s cause on their behalf at the national level. One respondent from a low-income country noted that such approaches were only successful if adults shared the view of the children, which was also evident in a high-income country participant’s reply, who emphasised that children’s voices had not been “… acted upon unless also grown-up professionals supported and advocated for the same views”. In other words, they, as adults, did not only carry the child’s voice further but could also garner respect through professional advocacy for the same point. Although policymakers had considered children and their rights, multi-layered gatekeeping was highlighted as a limiting factor regarding the access for both children’s voices and adult advocates on their behalf. Responses generally reflected limited efficacy in promoting children’s rights and voice, which led to the power of their narratives being limited, even barriered.

## 4. Discussion

This study addresses multidisciplinary child health-and-rights professionals’ experiences regarding safeguarding children’s UNCRC-protected rights to health and wellbeing during the initial COVID-19 pandemic. The survey reached professionals across eight well-defined world regions. We applied a rights-based lens informed by Farmer’s [13] call for more robust equity measures in global health policy and Singer’s [14] theory on syndemics in epidemic settings to demonstrate the consequences of mitigating policies and their possibility to enhance vulnerabilities of marginalised groups, in our case children [16,26]. Two overarching themes were generated. The first theme illuminated that the integration of children’s rights in governmental emergency response was deficient and not equitable. The second theme focused on organisational structure, guided by the core principles of the UNCRC as laid out in our proposed 5P framework, namely, participation, protection, provision, preparation, and power. The results show that children’s involvement in the pandemic response was lacking in all five domains but particularly low in the domains of preparation, participation, and power (Figure 1).

Applying the UNCRC as a tool highlights the still-existing gap between theory and practice [26]. It shows how children’s rights were left vulnerable during a crisis, along with the multifaceted modes of inequities which further impacted children on both international and local levels. These inequities were explored by putting children’s priorities and values in the foreground in an analysis of emergency policy alongside their rights to be heard. These inequities were frequently rooted in overt and tacit sociocultural narratives of children; children’s rights were subjugated to those of adults and justified by the rhetoric of emergency, pressure, and unprecedented times.

The COVID-19 pandemic has highlighted tendencies to relegate children’s rights during emergencies. Furthermore, it has exacerbated the need to reconsider the application of the UNCRC’s principles as a tool to advocate for children’s visibility and rights in governance to address the depth of inequalities among children. To address the importance of proactive preparation and power to children’s agency, we built on the UNCRC’s 3P framework [29,30]. We suggested expanding the convention’s articles to form a 5P framework as a conceptual and analytical tool (Table 1). The results expose the vast inequities children face between and within countries, the social contingencies of their visibilities and status as citizens, and the barriers faced in advocating for their rights.

By arranging the core principles of the UNCRC into 5Ps, we have demonstrated the lack of awareness of children’s right to participation, especially during the initial government emergency responses, as their particular circumstances and vulnerability was not accounted for [9,11]. However, consideration of their involvement was reported by survey participants to be more present at the grassroot levels. Further, they highlighted that the global emphasis on physical distancing did hamper children’s need for protection [18] and did not give sufficient attention to their vulnerability to domestic violence caused by school closures [10]. Levels of provisions were reported to have depended on the pre-existing robustness of the country’s healthcare and social system. Adding the preparation domain revealed the reactive approach to children’s growing need for support and intervention during the unfolding of the COVID-19 pandemic. This calls for a proactive approach to children’s needs in times of crises [26,39]. Finally, the power of children’s voices was limited, further showing children’s reliance on adults to champion their rights [21,40].

Although we consider children a marginalised group [41], its heterogeneity must be considered. Nonetheless, our analysis demonstrates the global lack of urgency and importance for children’s rights and needs during early pandemic responses. Evidence shows that a range of adverse effects were produced in countries of all economic levels, indicating that public health measures compounded pre-existing threats to survival that marginalised families confront daily, as in syndemics, with detrimental and disproportionate consequences [40,42]. Participants reported that early assumptions of children being at lower clinical risk than adults exposed to COVID-19 brought closer attention to the already media-depicted image of children as carriers of the disease but at limited risk of illness themselves. As Ciotti et al. [43] suggest, this is an important observation, as media can be considered a mirror to governing societal discourses and narratives. In other instances, the subjugation of child rights was tacitly framed by policymakers as enforced solidarity to protect more vulnerable elders. However, they should have generally paralleled this by robust oversight, planning, and leadership to re-instate reduced child rights at the earliest opportunity and protect children adequately within and beyond this unfolding and unprecedented situation.

Survey participants mentioned school closures and educational disruption as new inequalities that were created while pre-existing inequities were exacerbated. The importance of the school system as an equity measure for children has been reported; Betthäuser, Bach-Mortensen, and Engzell’s [44] meta-analysis shows that learning deficits, as a result of the pandemic, are socioeconomically dependent and more prevalent among children from deprived social backgrounds. Further, closing schools and moving education online caused a gaping and expanding digital division, isolating children from education and accessing means to report, seek help, and communicate with friends [23,24,45]. The divide was due to a lack of digital devices, variable digital literacy of parents and children, internet signal, electricity, finances, and appropriate study or private spaces. Whilst digital solutions provide many opportunities when approaching groups that are generally difficult to reach [46], they create barriers towards groups living without high-speed internet, finance for data, internet, digital literacy, language barriers, lack of privacy, and fear of such systems [47]. On another note, schools are often critical health facilities for children, bringing access to healthcare to their near environment [48]. Consequently, closing schools also barriered their access to healthcare and adult advocates who work there and support children daily [49].

The participants frequently mentioned resource access and allocation inequity due to direct or covert discrimination of various factors. These included race, ethnicity, gender, age, social class or immigration status, disability, and parents’ political views. As they described, children’s needs vary according to their socioeconomic contexts, and as they were left unaddressed, the corresponding policy measures cannot be considered equitable. As an example, children at further risk of harm, abuse, or exploitation, such as disabled children, received the double blow of enhanced vulnerability and limited use of rights-based angle in policies [8].

The inequities reported here harmonise with the existing literature on the various points of discrimination for children already further marginalised during the pandemic; Geweniger et al.’s [50] findings show that children of lower socioeconomic status in Germany during the pandemic were 60% more likely to be rated by their parents as suffering depression. Additionally, children with complex chronic diseases saw their support needs heightened. Although the pandemic led to children spending more time with their families, access to healthcare and social support for Brazilian families of children with Down syndrome was limited [51]. Another study from Sweden by Fäldt et al. [52] described loneliness in their paediatric participants due to disabled children having small social networks. They were, therefore, at risk of becoming socially isolated. A recent review [53] found that underlying inequities impacted children’s routine vaccination coverage, where children within populations already struggling with vaccination rates were more likely to be behind or have missed their vaccination. Missing out on vaccination might negatively impact global targets, such as the Global Vaccine Action Plan (GAVP), the Immunization Agenda 2030, and the Sustainable Developmental Goals (SDGs) [54,55].

All nations, bar the United States, have ratified the UNCRC, taking on legal obligations under international law [56]. National governments must prioritise and normalise child rights-informed leadership and structures. Straightforward child rights-informed ethical decision-making protocols for crises should be developed in consultation with children and their advocates. As the scientific community anticipates future pandemics, it is vital to consider government responses and subsequent policy and practice implications critically to evaluate the multiple human rights dimensions along with the inclusion of the expertise of child health-and-rights advocates. While many respondents discussed improvements in child rights inclusion on the policy level, we have yet to see policymakers fully embrace children’s views in future pandemic preparation. In other words, governments were generally unprepared for the impact COVID-19 would have on children. Despite the considerable knowledge gained thus far, the level of preparedness for future pandemic threats remains alarmingly inadequate [39]. Therefore, there is an urgent need to learn from experiences to date and optimise future leadership responses and pandemic preparedness with children’s rights taken into account.

If we are to emancipate children from “nobodisation” and empower them as agents, policymakers must improve pandemic preparedness and actively consider children’s rights. Still today, children’s access to their rights is contingent on the decisions and actions of adults. Therefore, child health-and-rights professionals are vital in safeguarding the UNCRC principles on levels of governance. Children *have* rights [4], which must be protected in all social and political spaces. For this to happen, a child rights-based approach is needed to inform policy.

One strength of the research is its contribution to the literature on the status of children’s rights in the initial emergency response to the COVID-19 pandemic. It shows that adult-centred policymaking quickly glossed over children’s rights to curb the rapidly unfolding pandemic. The 5P approach, presented here, is an innovative approach to examine the implementation of children’s rights across different settings, which all have in common to be safeguarded by the UNCRC. Furthermore, the study offers a comprehensive cross-country/continental comparison, highlighting specific cases from regions and countries, as reported by experienced child health-and-rights specialists in their respective settings. Nonetheless, the study has several limitations. The research team disseminated the survey via purposeful non-probabilistic snowball sampling among members of ISSOP, INRICH, and CHIFA, experienced child health-and-rights professionals, aiming for diversity rather than representativity. Another limitation is found in possible language barriers as the survey was in English, and there is a bias towards English-speaking participants. Further, the survey applies to the early pandemic responses, so the answers are only relevant within a specific timeframe and might have changed as the pandemic unfolded.

Further research is needed on how the different articles of the UNCRC are applied in daily work with children to uphold their rights as is outlined in the convention. It is essential to explore the impact of national crises on children, such as ongoing wars, conflicts, and migration, which threaten the health and wellbeing of children and their families. Guided by the 5P framework, relevant research themed around children in times of national crises might include the following research questions: *participation*: to what extent are children given the opportunities to express their views?; *protection*: to what extent are children’s rights given attention in country reports to the UN Committee on Rights of the Child?; *provision*: to what extent are refugee children provided with education and healthcare?; *preparation*: to what extent are disabled children’s rights to healthcare and education respected for their future development?; and *power*: to what extent do the voices of street and working children influence policy and practice on their behalf?

## 5. Conclusions

The early response to the COVID-19 pandemic exposed the lack of prioritisation of children’s rights in drafting emergency policy, rendering their social status even more vulnerable and their rights often dismissed despite child health-and-rights advocacy. Inclusion and prioritisation of children and their rights has failed on many levels because of a lack of standardised process and leadership authority vis-à-vis their rights. There is an urgent need to develop global and national child rights-informed ethical decision-making frameworks for pandemic and crisis scenarios where children and professional advocates are included to safeguard their UNCRC-protected human rights. Children’s meaningful participation and power in matters that concern them should be normalised and enabled in line with the evidence base. Dedicated leaders and government departments should champion and advocate for children’s participation and power and rely on existing resources for guidance. The 5P framework can help with assorting and naming the rights at most risk of being overlooked in a policy drafting and thus the most urgent to safeguard.

### Recommendations

As stated in the UNCRC, governments should respect children’s rights in future pandemic preparations and ensure they systematically include Child Rights Impact Assessment and Evaluation before and after policy decisions.

Child health-and-rights professionals applying a child rights-based lens must be present, active, and authoritative in national decision-making during crises.

Consult children in policy drafting and maintain a child-friendly approach that garners children’s voices and acts on their recommendations to safeguard their health and wellbeing in an emergency crisis.

Apply the 5P approach as an innovative tool for policymakers to ensure children’s participation is meaningful and not tokenistic. Further, to measure the protection of child rights in times of national crisis and how these measures can be included in the country report by the UN Committee on Rights of Child.

Health leadership should be astute and proactive in addressing myths and narratives around children’s personhood, health, and needs.

Governments should recognise the role of NGOs and grassroots organisations, and means should be established and welcomed for effective communication between those with expertise on the ground and those with knowledge or responsibility for high-level national oversight.

## Figures and Tables

**Table 1:** The ‘5P’ domains of social paediatric research, practice, action, advocacy, and key associated child rights articles are reflected in each domain.

Domain	Relevant Articles in the UNCRC by Domain
PARTICIPATION of children	Art. 3 Best interests Art. 5 Parental responsibility but also recognising evolving capacities of the child Art. 12 Child’s view respected
PROVISION for children’s health and developmental needs	Art. 4 and 42 Governments must promote and protect child rights Art. 6 Life, survival, and development Art. 13 and 17 Ability to access information Art. 18 Both parents share responsibility; the state must provide services Art. 23 Disabled children supported to live full life with dignity Art. 24 Health resources (clean water, food, environments) and healthcare Art. 28 Right to education Art. 31 Play and leisure Art. 39 Special support after trauma
PROTECTION of children from harms	Art. 2 Non-discrimination Art. 3 Best interests Art. 6 and 27 Life, survival, and development Art. 17 Access to age-appropriate health-promoting information and protection from harmful media content Art. 19 Protection from violence, abuse, neglect Art. 22 Refugee children are protected and assisted to enjoy their rights Art. 24 Health resources (clean water, food, environments) and healthcare Art. 32 Protection from economic and labour exploitation Art. 33 Protection from involvement with illegal drugs Art. 34 Protection from sexual exploitation Art. 35 Protection from trafficking
PREPARATION for future child health crises	Art. 4 Governments must promote and protect children’s rights Art. 6 Life, survival, and development Art. 12 Child’s view respected Art. 24 Health resources (clean water, food, environments) and Healthcare Art. 28 Right to education
POWER authority of children’s voices, which requires meaningful participation	Art. 3 Best interests Art. 12 Child’s view respected

## Data Availability

The data presented in this study are available on reasonable request from the corresponding author. The data are not publicly available due to confidentiality laws under the European GDPR Act 2018.

## Supplementary Materials

The following supporting information can be downloaded at: https://www.mdpi.com/article/10.3390/children10101670/s1, File S1: Child Rights During COVID-19 Survey Questions .
