# Supplementary material for: Child Rights during the COVID-19 Pandemic: Learning from Child Health-and-Rights Professionals across the World
Source: Children (Basel). 2023 Oct 9;10(10):1670. doi: 10.3390/children10101670 (PMC10605735; doi:10.3390/children10101670)
Supplement: Supplementary file 1 [file children-10-01670-s001.zip › children-2622094-supplementary.pdf]

What has happened to Child Rights & Health during COVID-19? ISSOP & INRICH* Social Paediatrics & Child Health Survey

Your experiences as a child health professional during the COVID-19 pandemic are important.

In this survey, we want to hear your ideas, observations and concerns about *child rights and child health* in your nation during the pandemic.

Please tell us about the child rights and child health issues you know about from your work. Include any *changes* in the pandemic response that have affected child rights and health. You may add links to policy or research papers in your answers, but *do not worry* if they are not yet available.

Who should complete this survey?

Social paediatricians and child health professionals working in any nation(s).

Where will my answers go?

Your answers will go to Dr Laura C N Wood and the Northern Europe ISSOP & INRICH research group chaired by Professor Geir Gunnlaugsson, Global Health, University of Iceland.

The initial purpose of this survey is to help ISSOP & INRICH understand how child rights have been affected by the COVID-19 pandemic. The answers will be coded by volunteers from the ISSOP Northern Europe team and the results shared among ISSOP and INRICH.

We would like to keep the possibility open for this project to lead to further research and publications. In this case, all data leading to individual identification will be omitted and results analysed on national and/or regional levels only.

In completing this survey, you are consenting for your responses to be stored securely for future research by ISSOP & INRICH.

Do you have a question or concern?

Please email Dr Laura C N Wood
laura.wood@lancaster.ac.uk

THANK YOU!

* ISSOP is the International Society for Social Paediatrics and Child Health, https://www.issop.org/
INRICH is the International Network for Research in Inequalities in Child Health, https://inrichnetwork.org/

OK

1. What nation and region do you work in as a child health professional? Please use this nation and region for all of your answers (ie Germany - Bavaria, South Africa - KwaZulu Natal)

2. Have Child Rights been considered in your national COVID-19 response?

- ◯ YES
- ◯ PARTLY (only some child rights and/or only at some times in the pandemic)
- ◯ NO
- ◯ I DO NOT KNOW

PLEASE EXPLAIN YOUR ANSWER using at least one example from your nation (include the timing or stage of the pandemic where relevant):

3. Has the COVID-19 response in your nation been EQUITABLE (right, just and fair) to all children?

- ◯ YES
- ◯ PARTLY (only right, just and fair for some children)
- ◯ NO
- ◯ I DO NOT KNOW

PLEASE EXPLAIN YOUR ANSWER using at least one example from your nation (include the timing or stage of the pandemic where relevant):

4. Have children in your nation PARTICIPATED (taken part) in decisions about COVID-19 responses by telling decision-makers their views and needs? Examples: through surveys for children, research focus groups, children's advisory groups, school-based decision making, NGO or charity groups etc.

- ◯ YES
- ◯ PARTLY (only some children or groups of children participated)
- ◯ NO
- ◯ I DO NOT KNOW

PLEASE EXPLAIN YOUR ANSWER using at least one example from your nation (include the timing or stage of the pandemic where relevant):

5. Have decision-makers in your nation ACTED ON the views of children who have participated, giving children's voices POWER (authority and respect)?

- ◯ YES
- ◯ PARTLY (acted partly on children's views and/or acted only on the views of particular groups of children)
- ◯ NO
- ◯ I DO NOT KNOW

PLEASE EXPLAIN YOUR ANSWER using at least one example from your nation (include the timing or stage of the pandemic where relevant):

6. Have children been PROTECTED from harm (biological, physical, psychological, mental, emotional, and/or developmental harm) through COVID-19 responses in your nation? Examples: vaccination schedules, child protection, NGO and charity activities etc.

- ◯ YES
- ◯ PARTLY (only some children have been protected and/or only protected in some ways)
- ◯ NO
- ◯ I DO NOT KNOW

PLEASE EXPLAIN YOUR ANSWER using at least one example from your nation (include the timing or stage of the pandemic where relevant):

7. Have children been given the PROVISION they need for good health and development during the COVID-19 response in your nation? Examples: provision of shelter, nutritious food, clean water, education, healthcare etc.

- ◯ YES
- ◯ PARTLY (only some children and/or some provisions were given)
- ◯ NO
- ◯ I DO NOT KNOW

PLEASE EXPLAIN YOUR ANSWER using at least one example from your nation (include the timing or stage of the pandemic where relevant):

8. Has your nation shown PREPARATION for the health and developmental needs of children in the future?
Examples: planning for increased mental health problems, future education planning, new child health research groups, extra training for health professionals, planning for future pandemics etc.

- ◯ YES
- ◯ PARTLY (some actions taken for the future)
- ◯ NO
- ◯ I DO NOT KNOW

PLEASE EXPLAIN YOUR ANSWER using at least one example from your nation (include the timing or stage of the pandemic where relevant):

9. During this pandemic, have you learnt something about child rights and child health that you wish to share?

- ◯ YES
- ◯ NO
- ◯ I DO NOT KNOW YET

If YES, please describe your important learning points here:

10. Please record your PROFESSION (ie nurse, doctor, researcher, social worker etc.)
